# Emotional Dysregulation-Mediated Associations Between Guilt Proneness, Shame Proneness, and Internet Gaming Disorder Among Chinese University Students: Cross-Sectional Survey

**DOI:** 10.2196/74052

**Published:** 2025-09-05

**Authors:** Yanqiu Yu, Xin Su, Ying Yang, Haifeng Xue, Bo Liu, Yuqi Sun, Ruxin Wang, Xinxin Mo, Hongye Luo, Lijuan Li, Xianzhang Tian, Yanjie Yang, Zhengxue Qiao, Liping Li, Tong Xie, Siman Li, Lijing Liu, Yucheng Zhang, Joseph T F Lau

**Affiliations:** 1 School of Public Health, Fudan University, Shanghai, China; 2 School of Public Health, Baotou Medical College, Inner Mongolia University of Science & Technology, Baotou, China; 3 Department of Nuclear Medicine, The First Affiliated Hospital of Baotou Medical College, Inner Mongolia University of Science and Technology, Baotou, China; 4 School of Public Health, Qiqihar Medical University, Qiqihar, China; 5 The Second Affiliated Hospital of Qiqihar Medical University, Qiqihar, China; 6 School of Mental Health, Wenzhou Medical University, Wenzhou, China; 7 Research Centre for Regenerative Medicine, Guangxi Medical University, Nanning, China; 8 Information and Management School, Guangxi Medical University, Nanning, China; 9 School of Public Health, Dali University, Dali, China; 10 Health Science Center, Dali University, Dali, China; 11 Psychology and Health Management Center, Harbin Medical University, Harbin, China; 12 School of Public Health, Shantou University, Shantou, China; 13 Zhejiang Provincial Clinical Research Center for Mental Disorders, The Affiliated Wenzhou Kangning Hospital, Wenzhou Medical University, 1 Shengjing Road, Lucheng District, Wenzhou, 325000, China, 86 13143882252

**Keywords:** shame proneness, guilt proneness, emotional dysregulation, internet gaming disorder, medical students, China

## Abstract

**Background:**

Guilt proneness and shame proneness are interconnected yet distinct personality traits that are gaining attention in addiction research. However, studies examining their differential associations with internet gaming disorder (IGD) and mediation mechanisms explaining these associations remain scarce. Theoretical and empirical evidence suggests that emotional dysregulation could be a potential mediator of the associations between guilt proneness and shame proneness and IGD.

**Objective:**

This study aimed to investigate the associations between guilt proneness in cognitive (guilt-negative behavior-evaluations) and behavioral (guilt-repair) domains and shame proneness in cognitive (shame-negative self-evaluations) and behavioral (shame-withdrawal) domains and IGD, as well as related mediation mechanisms via emotional dysregulation.

**Methods:**

A multicenter, cross-sectional, anonymous online survey was conducted among medical undergraduate students in seven Chinese cities (Wenzhou, Dali, Nanning, Harbin, Baotou, Qiqihar, and Shantou) from December 2023 to February 2024. In total, 12,912 invitations were sent out, of which 8522 eligible cases were included; the mean response rate was 71.0%. The 9-item DSM-5 IGD Checklist was used to screen for IGD cases; the 16-item Guilt and Shame Proneness Scale was used to assess guilt and shame proneness; the Cognitive Emotional Regulation Questionnaire was used to assess emotional dysregulation. Univariate logistic regression analysis was conducted to examine the associations between background factors and IGD. Structural equation modeling (SEM) was performed to test the mediation mechanism, with the adjustment of background factors.

**Results:**

Of all participants, the prevalence of IGD was 7.5%. Background factors of male sex (vs female, OR 2.78, 95% CI 2.36, 3.28) and self-reported poor household financial situation (vs good, odds ratio [OR] 1.96, 95% CI 1.51, 2.55) were significantly associated with a higher risk of IGD; the associations involving study city, year of study, study major, and origin (residency) of students were statistically nonsignificant. SEM showed that shame proneness in both cognitive (*β*=.29, 95% CI 0.26, 0.33) and behavioral (*β*=.20, 95% CI 0.18, 0.22) domains and emotional dysregulation of rumination, catastrophizing, and self-blame (*β*=.36, 95% CI 0.32, 0.40) were positively associated with IGD, while guilt proneness in both cognitive (*β*=−.08, 95% CI −0.12 to −0.03) and behavioral domains (*β*=−0.10, 95% CI −0.14 to −0.06) was negatively associated with IGD. Furthermore, emotional dysregulation partially mediated the association between guilt proneness and shame proneness in both domains and IGD (mediation effect size ranged from 22.0% to 45.8%).

**Conclusions:**

This study observed relatively high prevalence of IGD among medical undergraduate students in China. Furthermore, the associations between shame and guilt proneness and IGD were differential. Shame proneness and guilt proneness in cognitive and behavioral domains were both directly and indirectly (via emotional dysregulation) associated with IGD, suggesting that future intervention studies may reduce maladaptive shame proneness or shift from shame towards adaptive guilt to reduce the risk of IGD.

## Introduction

Internet gaming disorder (IGD) is an emerging global public health concern; in 2013, it was included in the Fifth Edition of the Diagnostic and Statistical Manual of Mental Disorders (DSM-5) as a mental condition warranting further research [1]. In 2018, along with offline video gaming, IGD was officially included in the 11th Revision of the International Classification of Diseases (ICD-11) as a mental disorder, which is characterized by behavioral symptoms of impaired control over gaming, prioritizing gaming over other activities to the extent that gaming becomes the dominant daily activity, continuation of or increase in gaming despite having negative consequences, and significant impairments to academic, social, or other important functions [2]. China ranks first in the number of internet gamers and the revenue of the gaming industry worldwide. As of June 2023, the number of internet gamers reached 550 million, with an increased rate of 5.4% [3]. Young adults, in particular university students, face various challenges related to academic performance, social adjustment, and career uncertainty [4], and hence are vulnerable to IGD. A meta-analysis of 155 studies reported that the pooled prevalence of IGD among young adults (aged 18‐28 y; 10.4%) was higher than that among adolescents (aged 8‐18 y; 8.8%) [5]; the prevalence ranged from 5.5% to 14.8% among Chinese university students [6-8]. IGD could result in various negative consequences among university students, including poor academic performance [9] and lower levels of self-esteem, social support, and life satisfaction [10]. It is hence warranted to understand better the determinants of IGD and relevant mechanisms to facilitate effective prevention and treatment among university students.

### Associations Between Guilt or Shame Proneness and IGD

Both guilt and shame are emotional experiences that arise in response to potential conflicts with personal or public moral standards and social norms [11]. They are closely related, yet distinct self-conscious emotions. Guilt refers to the feelings of remorse about a specific behavior (eg, “I did something wrong”), which is related to one’s empathetic concerns for others and action tendencies to repair the harm inflicted on others; it is in general depicted as an adaptive response, resulting in prosocial behaviors, including making confession and apology as well as taking reparative and corrective actions [12-14]. However, notably, researchers argued that guilt could become maladaptive if it were excessively strong or lasted longer than the situation warranted [15]. In contrast, shame involves a negative evaluation of oneself as a whole (eg, “I am a bad person”) [13,14,16]. Despite that the occasional feelings of shame might be normal [15], shame is in general depicted as a maladaptive response, leading to two types of anti-social behaviors: (1) the tendency to avoid any shame-inducing events and (2) defensive aggression towards others to shift blame for such events [13,14,16].

Theoretical (eg, the Interaction of Person-Affect-Cognition-Execution model of internet addiction) and empirical evidence highlight the predictive effects of personality traits on IGD [17,18]. Guilt and shame proneness are 2 typical personality traits with emerging scholarly attention in the field of addiction research; they refer to one’s tendency toward experiencing guilt and shame, respectively [19]. The well-established Guilt and Shame Proneness Scale distinguished between cognitive and behavioral responses related to guilt and shame proneness [19]. Accordingly, guilt proneness comprises two domains of negative behavior evaluations (Guilt-Negative Behavior Evaluations; eg, feeling terrible after telling a lie) and repair action tendencies (Guilt-Repair; eg, making compensations), and shame proneness comprises two domains of negative self-evaluations (Shame-Negative Self-Evaluations; eg, feeling like a bad person) and withdrawal action tendencies (Shame-Withdraw; eg, retreating from others) [19]. Corroborating with the above conceptualization of guilt and shame, the extant literature reported that guilt proneness was associated with positive attributes such as prosocial and approach orientation [12,20] while shame proneness was associated with negative outcomes, including emotional difficulties, social withdrawal, and aggression toward others [12,16]. In the context of digital addiction, to our knowledge, there were only four relevant studies, and the findings were mixed. One study reported the positive association between shame and internet addiction among Iranian university students [21]; 2 reported that shame was positively associated with problematic social media use which was also negatively associated with guilt [22,23]; yet, another one reported inconsistent findings that interpersonal guilt was positively associated with IGD [24]. More studies are hence needed to elucidate the associations between guilt and shame proneness and IGD.

### Emotional Dysregulation as a Mediator Between Guilt or Shame Proneness and IGD

Emotional dysregulation refers to the difficulties in managing and responding to emotional experiences in an adaptive way; it involves a persistent pattern of emotional instability and an inability to effectively regulate emotions [25]. Theoretically, the process model of emotional regulation postulates that individuals tend to use adaptive or maladaptive strategies to manage and respond to their emotional experiences, which would enhance or diminish positive emotions, leading to favorable or unfavorable outcomes [26]. As aforementioned, guilt and shame are emotional responses related to perceived wrongdoing or failure [11,12], which may trigger emotional regulation or dysregulation strategies. Empirical evidence also reported positive associations between guilt/shame and difficulties in emotional regulation [24]. Therefore, emotional dysregulation could be a potential mediator of the association between guilt and shame proneness and IGD; that is, guilt and shame proneness would be significantly associated with emotional dysregulation, which would in turn be positively associated with the risk of IGD.

Specifically, rumination, catastrophizing, and self-blame are representative of emotional dysregulation [25]. Rumination refers to repetitive thoughts about the distressing aspects of a situation; catastrophizing refers to the cognitive distortion of imagining the worst possible outcome in a situation; self-blame refers to the tendency to hold oneself responsible for negative outcomes [25]. Understandably, guilt and shame are self-conscious emotions that involve intense self-reflection and evaluation, which could inherently trigger emotional dysregulation strategies of rumination (the repetitive focus on one’s distress driven by similar self-focused attention), catastrophizing (the tendency to interpret events in a negatively exaggerated manner), and self-blame. Self-blame manifested as (1) behavioral self-blame, ie, attributions directed toward modifiable behaviors and tied to guilt, and (2) characterological self-blame, that is, attributions directed toward the global self and more closely related to shame [27,28]. Notably, while adaptive guilt may prompt reparative actions and yield favorable outcomes [12-14], excessive or maladaptive guilt might lead to adverse consequences [15]. Thus, the associations between guilt proneness and emotional dysregulation might be either positive or negative. Reviews have also reported mixed results involving positive, negative, and nonsignificant associations between guilt and rumination, catastrophizing, and self-blame [28,29]. In contrast, existing literature more consistently reported strong and positive associations between shame and these emotional dysregulation strategies [28,29]. It was hence hypothesized that shame proneness would be positively associated with rumination, catastrophizing, and self-blame.

In addition, emotional dysregulation of rumination, catastrophizing, and self-blame were known determinants of IGD [30,31], as internet gaming could serve as an easily accessible and effective tool to modulate and avoid those negative moods and emotions [32,33]. It was hence assumed that guilt or shame proneness would be associated with emotional dysregulation of rumination, catastrophizing, and self-blame, which would in turn increase the risk of IGD. Our literature search located only 2 relevant studies. One study found that emotional dysregulation mediated the association between interpersonal guilt and IGD [24], while the other one reported that difficulties in emotional regulation mediated between shame or guilt and problematic social media use [22].

### Study Objectives

This study aimed to investigate the prevalence of IGD and its associations with guilt proneness, shame proneness, and 3 types of emotional dysregulation (rumination, catastrophizing, and self-blame) among undergraduate students in 7 Chinese cities. The mediation effects of emotional dysregulation on the associations between guilt and shame proneness and IGD were tested. It was hypothesized that guilt and shame proneness would be significantly associated with the latent variable of emotional dysregulation (rumination, catastrophizing, and self-blame), which would in turn be positively associated with IGD. Specifically, the associations between guilt proneness and emotional dysregulation were hypothesized to be either positive or negative, while those between shame proneness and emotional dysregulation were hypothesized to be positive.

## Methods

### Participants and Data Collection

A multicenter cross-sectional survey was conducted among undergraduate medical students from 7 universities in 7 cities in China from December 2023 to February 2024, including Wenzhou (east China), Dali (southwest), Nanning (southwest), Harbin (northeast), Qiqihar (northeast), Baotou (north), and Shantou (south). Inclusion criteria for participants included (1) age of 18 or above, (2) full-time undergraduate students, (3) being able to read and understand Chinese, and (4) being willing to participate in this study and provide informed consent.

This study used stratified cluster sampling; the sampling and data collection procedures were consistent in all the participating medical colleges and universities. Undergraduate medical education takes 5 years in China, and students are grouped into classes based on their majors. In each grade of the participating colleges or universities, the classes with a major in clinical medicine versus nonclinical medicine (eg, public health) were randomly selected in a ratio of 1:1, and all undergraduate students from the selected classes were invited to join the study. The coordinating teachers and student helpers sent the students an invitation letter, a hyperlink with access to a structured questionnaire, and several reminders via a WeChat group used for in-class communication. It was clearly stated in the invitation message and cover page of the questionnaire that the participation was voluntary and anonymous, and that refusal would not generate any negative consequences. Students were requested to endorse a question indicating informed consent; no signature was requested to maintain anonymity. The questionnaire took about 30 minutes to complete.

A total of 12,912 invitations were sent out; 9163 returned the completed questionnaires (a response rate of 71.0%), among which 611 were excluded due to failure in passing the quality check (eg, the completion time was less than three minutes). The final sample size used for data analysis was 8552.

### Ethical Considerations

Participants were clearly informed by the coordinators and in both the invitation letter and the cover page of the questionnaire about the purpose, content, and voluntary and anonymous nature of this survey. After submitting the completed questionnaires, the students could voluntarily join a lottery draw that offered 30 cash prizes in total (4 of 100 RMB, 6 of 50 RMB, and 20 of 20 RMB); no other incentives were involved. No signatures were requested from the participants to maintain anonymity. This study was approved by the ethics committee of the corresponding author’s affiliation (Ref No. 2023‐017).

### Measurements

(Refer to MultimediaAppendices 1  2 for the Questionnaires in English and in Chinese, Respectively)

#### Background Information

Such information was collected, including age, sex, city, year of study, major, whether being a local student, and self-rated household financial situation.

#### IGD

It was assessed by using the 9-item DSM-5 IGD Checklist, which assessed the presence of nine IGD symptoms in the past 12 months, including preoccupation, withdrawal symptoms, tolerance, inability to control gaming, continuation of gaming despite negative consequences, deception of gaming time, prioritization of gaming over other hobbies or interests, using gaming as a way to escape pressures or negative mood, significant losses in interpersonal relationships or career due to gaming [1]. The items were rated on binary response options (0=No, 1=Yes). Those who endorsed 5 or more symptoms would be classified as IGD cases. The Chinese version of the checklist and its cut-off value (≥5 of 9) have been validated among Chinese adults [34,35]. The Cronbach α of the checklist in this study was .86.

#### Guilt and Shame Proneness

They were assessed by using the 16-item GASP [19]; this scenario-based scale aims to assess individual differences in the propensity to experience guilt and shame across a range of personal transgressions, and it comprises four subscales of Guilt-NBE (Negative behavior-evaluations), Guilt-repair, Shame-NSE (Negative self-evaluations), and Shame-withdrawal as mentioned in Introduction [19]. The items were rated on a 7-point Likert scale (1=very unlikely to 7=very likely), with higher scores indicating higher levels of respective guilt/shame proneness. The scale underwent a rigorous translation process involving both English-to-Chinese and Chinese-to-English translations by two experienced bilingual researchers; any discrepancies during this process were resolved with the assistance of another bilingual senior researcher (ie, the corresponding author of this study). Confirmatory factor analyses confirmed the 4-factor structure of the GASP in this study, with all factor loadings over 0.54 (all *P*<.001) and satisfactory model fit indices [Comparative Fit Index (CFI)=0.93, Root Mean Square Error of Approximation (RMSEA)=0.06, and Standardized Root Mean Squared Residual (SRMR)=0.06]. Furthermore, the Cronbach α of the four subscales ranged from .70 to .84 in this study, indicating good internal consistency.

#### Emotional Dysregulation

It was assessed by using the three 2-item subscales of rumination, catastrophizing, and self-blame of the 18-item Cognitive Emotion Regulation Questionnaire [25], which has been validated among Chinese university students with good psychometric properties [36]. Rumination was assessed by understanding the frequency of recalled past feelings; catastrophizing was assessed by the rate of focusing on the negative side of past events; self-blame was assessed by the tendency of individuals to attribute blame to themselves for negative occurrences. The items were rated by using 5-point Likert scales (1=almost never to 5=always); higher scores indicated higher levels of respective emotional dysregulation responses. The Cronbach α of the 3 subscales ranged from .78 to .93; the subscales have also been commonly used in previous studies [37,38].

### Statistical Analysis

IGD was used as the binary dependent variable in this study. Univariate logistic regression analyses were conducted to examine the significance of the background factors of IGD; crude odds ratio (ORc) and respective 95% CIs were reported. Structural equation modeling (SEM), with the estimator of Weighted Least Squares with Mean and Variance, was conducted to examine the mediation effect of emotional dysregulation on the associations between guilt/shame proneness in both domains and IGD. The Weighted Least Squares Mean and Variance adjusted (WLSMV) estimator is appropriate for analyzing models with categorical dependent variables (including the binary outcome in this study), as it provides robust standard errors and correct *χ*^2^ test statistics under violations of normality assumptions [39]. Background factors were specified as predictors of both the mediators and the dependent variable in SEM to statistically control for their potential confounding effects [40]. The latent variable of emotional dysregulation was generated from the subscale scores of rumination, catastrophizing, and self-blame. Satisfactory model fit indices of the SEM included CFI≥0.90, RMSEA≤0.80, and SRMR≤0.08 [39]. Following MacKinnon’s approach [40], the indirect effects of mediation were calculated as the product of the path from the independent variable to the mediator (a) and the path from the mediator to the binary dependent variable (b). The direct effect was represented by the path from the independent variable to the binary dependent variable (c’), and the total effect was the sum of indirect and direct effects (ie, a×b+c’). The mediation effect size (ie, the proportion of the total effect explained by the mediator) was calculated by dividing the indirect effect by the total effect. The SEM was conducted by using Mplus 8.0, while the other analyses were conducted by SPSS (version 23.0; IBM Corp). Statistical analysis was defined as a two-tailed *P* value<.05.

### Sample Size Calculation

The sample size planning was based on the key statistical method of SEM, which requires a minimum of ten cases per observed variable [41]. In this study, the smallest participant-to-variable ratio was 84:1, observed in Shantou city (n=674). Therefore, the sample size was considered adequate across all study cities.

## Results

### Descriptive Statistics

**Figure 1.**
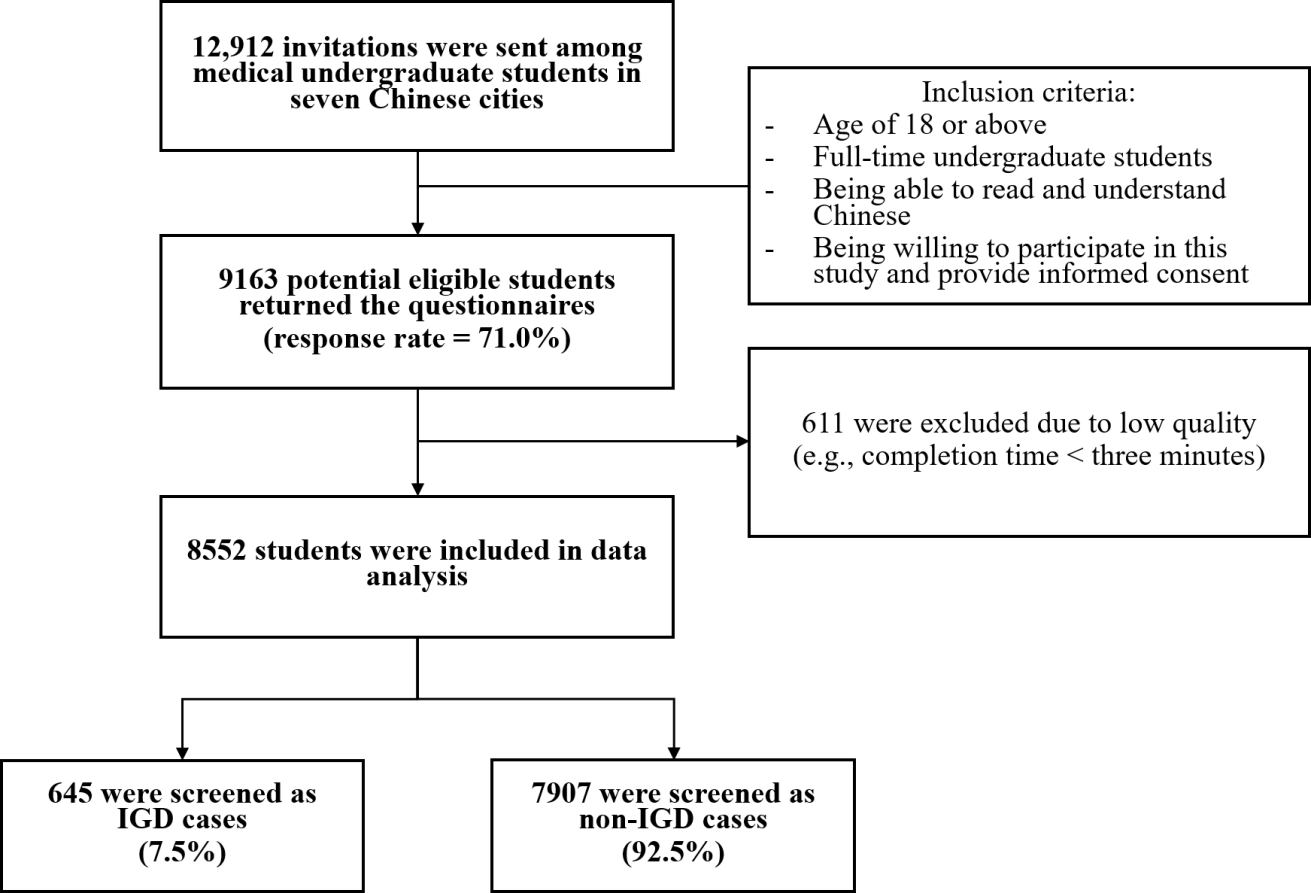
Flowchart of participant recruitment. IGD: internet gaming disorder.

The flowchart of participant recruitment is presented in Figure 1. Of all participants, 15.5% (1327/8552), 10.2% (875/8552), 11.1% (951/8552), 9.9% (845/8552), 24.6% (2100/8552), 20.8% (1780/8552), and 7.9% (674/8552) were recruited from Wenzhou, Dali, Nanning, Harbin, Baotou, Qiqihar, and Shantou, respectively. The mean (SD; range) age was 19.9 (1.5; 18‐30) years; over half were female (5470/8552, 64.0%); about one-third majored in clinical medicine (2855/8552, 33.3%); the majority were Year 1 to Year 3 undergraduates (6911/8552, 80.8%) and nonlocal students (7402/8552, 86.5%); nearly one-fifth self-reported poor household financial situation. The prevalence of IGD was 7.5% (645/8552) (Table 1).

**Table 1.** Participants’ characteristics (N=8552).

Background information	Participants (N=8552), n (%)
City of study	
Wenzhou	1327 (15.5)
Dali	875 (10.2)
Nanning	951 (11.1)
Harbin	845 (9.9)
Baotou	2100 (24.6)
Qiqihar	1780 (20.8)
Shantou	674 (7.9)
Sex	
Female	5470 (64.0)
Male	3082 (36.0)
Year of study	
Year 1	2861 (33.5)
Year 2	2096 (24.5)
Year 3	1954 (22.8)
Year 4	1178 (13.8)
Year 5	463 (5.4)
Study major	
Clinical medicine	2855 (33.4)
Others (eg, public health and pharmacy)	5697 (66.6)
Origin (residency) of students	
City of study	1150 (13.4)
City of the study province	4964 (58.0)
City outside the study province	2438 (28.5)
Self-rated household financial situation	
Good/very good	1437 (16.8)
Moderate	5600 (65.5)
Poor/very poor	1515 (17.7)
Internet gaming disorder	
No	7907 (92.5)
Yes	645 (7.5)

The mean (SD) scores were 21.4 (5.6) for guilt-NBE, 21.2 (5.5) for guilt-repair, 19.9 (5.5) for shame-NSE, and 16.1 (4.9) for shame-withdraw (all range=4 to 28). The mean (SD) scores were 6.0 (2.0) for rumination, 4.6 (2.2) for catastrophizing, and 4.8 (2.1) for self-blame (all range=2 to 10). These statistics were not tabulated.

### Background Factors of IGD

Table 2 showed that male students (vs female, crude odds ratio [ORc]=2.78, 95% CI: 2.36, 3.28) and those from self-rated poor household financial situation (vs good, ORc=1.96, 95% CI: 1.51, 2.55) and study city of Nanning (vs Wenzhou, ORc=1.37, 95% CI: 1.04, 1.80) were at higher risk of IGD, while those from study cities of Harbin (vs Wenzhou, ORc=0.66, 95% CI: 0.47, 0.93) and Baotou (vs Wenzhou, ORc=0.49, 95% CI: 0.37, 0.65) were at lower risk of IGD. The associations involving other background factors were statistically nonsignificant.

**Table 2.** The associations between background factors and internet gaming disorder (IGD) (n=8552).

Background factors	IGD	
	ORc^a^ (95% CI)	*P* value
City of study		
Wenzhou	1.0 (Reference)	—^b^
Dali	1.07 (0.79, 1.43)	.68
Nanning	1.37 (1.04, 1.80)	.03
Harbin	0.66 (0.47, 0.93)	.02
Baotou	0.49 (0.37, 0.65)	<.001
Qiqihar	0.83 (0.64, 1.08)	.16
Shantou	1.08 (0.78, 1.50)	.65
Age	1.04 (0.99, 1.09)	.16
Sex		
Female	1.0 (Reference)	—
Male	2.78 (2.36, 3.28)	<.001
Year of study		
Year 1	1.0 (Reference)	—
Year 2	1.22 (0.99, 1.51)	.06
Year 3	1.21 (0.98, 1.51)	.08
Year 4	0.88 (0.67, 1.16)	.37
Year 5	1.31 (0.92, 1.87)	.13
Study major		
Clinical medicine	1.0 (Reference)	—
Others (eg, public health and pharmacy)	1.08 (0.91, 1.29)	.37
Origin (residency) of students		
City of study	1.0 (Reference)	—
City of the province of study	1.02 (0.80, 1.30)	.87
City outside the province of study	1.04 (0.79, 1.35)	.80
Self-rated household financial situation		
Good	1.0 (Reference)	—
Moderate	1.01 (0.80, 1.27)	.97
Poor	1.96 (1.51, 2.55)	<.001

a ORc: crude odds ratio.

b Not applicable.

### Mediation Analysis by SEM

**Figure 2.**
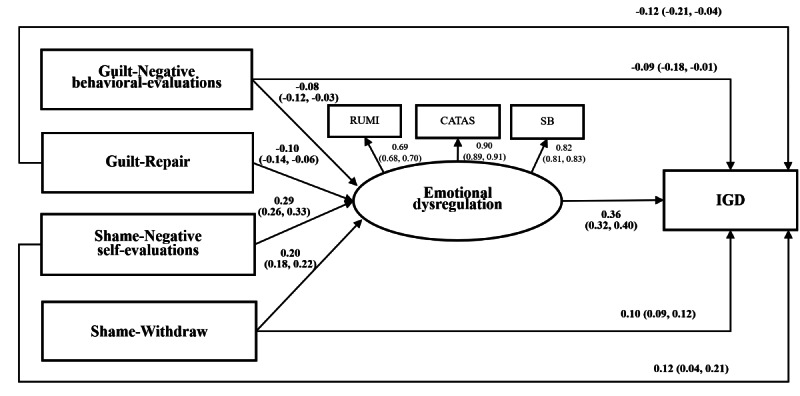
Mediation analysis by structural equation modelling (Standardized coefficients and their respective 95% CIs were reported. IGD: internet gaming disorder; RUMI: rumination; CATAS: catastrophizing; SB: self-blame. The model was adjusted for background factors, including city, age, sex, year of study, study major, origin of students, and self-rated household financial situation).

The correlations among the studied variables are presented in Multimedia Appendix 3. Figure 2 and Table 3 present the mediation effects of the latent variable of emotional dysregulation on the associations between guilt and shame proneness and IGD, after adjusting for background factors. The model demonstrated satisfactory model fit indices, ie, CFI=0.91, RMSEA=0.03, SRMR=0.04. Guilt proneness in both cognitive and behavioral domains was negatively associated with the latent variable of emotional dysregulation, which in turn was positively associated with IGD. Both guilt-NBE and guilt-repair had significant direct effects and indirect effects on IGD, indicating partial mediation via emotional dysregulation (effect size=22.9% and 22.0%, respectively). In contrast, shame proneness in both cognitive and behavioral domains was positively associated with the latent variable of emotional dysregulation, which in turn was positively associated with IGD as aforementioned. Both shame-NSE and shame-withdrawal had significant direct effects and indirect effects on IGD, indicating partial mediation via emotional dysregulation (effect size=45.8% and 41.3%, respectively).

**Table 3.** Mediation analysis by structural equation modelling.

Paths in the mediation model	*β* (95% CI)^a^	*P* value	Mediation effect size (%)
Structural paths			
Guilt-NBE^b^ → Emotional dysregulation	−.08 (−0.12 to −0.03)	.001	—^c^
Guilt-Repair → Emotional dysregulation	−.10 (−0.14 to −0.06)	<.001	—
Shame-NSE^d^ → Emotional dysregulation	.29 (0.26 to 0.33)	<.001	—
Shame-Withdrawal → Emotional dysregulation	.20 (0.18 to 0.22)	<.001	—
Emotional dysregulation → IGD^e^	.36 (0.32 to 0.40)	<.001	—
Guilt-NBE → IGD	−.09 (−0.18 to −0.01)	.04	—
Guilt-Repair → IGD	−.12 (−0.21 to −0.04)	.004	—
Shame-NSE → IGD	.12 (0.04 to 0.21)	.003	—
Shame-Withdrawal → IGD	.10 (0.06 to 0.15)	<.001	—
Indirect paths			
Guilt-NBE → Emotional dysregulation → IGD	−.03 (−0.04 to −0.01)	.001	22.9
Guilt-Repair → Emotional dysregulation → IGD	−.04 (−0.05 to −0.02)	<.001	22.0
Shame-NSE → Emotional dysregulation → IGD	.10 (0.09 to 0.12)	<.001	45.8
Shame-Withdrawal → Emotional dysregulation → IGD	.07 (0.06 to 0.08)	<.001	41.3

a The model was adjusted for background factors, including city, age, sex, year of study, study major, origin of students, and self-rated household financial situation.

b NBE: negative behavior evaluations.

c Not applicable.

d NSE: negative self-evaluations.

e IGD: internet gaming disorder.

## Discussion

### Principal Findings

This multicenter study aimed to explore the associations between guilt and shame proneness and IGD via emotional dysregulation via rumination, catastrophizing, and self-blame among Chinese undergraduate students. The results indicated distinct associations: guilt proneness in both domains was negatively associated with both emotional dysregulation and IGD, while shame proneness in both domains showed positive associations with these outcomes. In addition, emotional dysregulation partially mediated the associations between guilt/shame proneness and IGD. These findings provide valuable insights into future research and the development of targeted interventions addressing IGD.

### Comparison to Prior Work

This study observed the prevalence of IGD of 7.5% among medical undergraduates in seven Chinese cities, which was comparable to that among medical students in India (7.2%) [42] and among general university students in Macao, China (7.4%) [8]; all the comparisons were made based on the same measurement tool and cut-off value of IGD. The relatively consistent IGD prevalence implies common gaming patterns among Asian university students, particularly in demanding academic environments. Such an assumption was supported by the nonsignificant associations between study major or study year and IGD. Medical undergraduates undergoing intense academic stress may have similar gaming patterns irrespective of majors and grades. Nonetheless, compared to Wenzhou, Dali, Qiqihar, and Shantou, students in Nanning exhibited higher risk of IGD while those in Harbin and Baotou exhibited a lower risk. These city-related differences may be explained by the variations in social, economic, and cultural environments. Factors such as local attitudes towards gaming, availability of recreational options, and broader community context (eg, access to mental health resources) may influence students’ gaming behavior and, consequently, the prevalence of IGD. In addition, students of male sex and self-rated poor household financial situation were at higher risk of IGD in this study, which corroborates previous findings in the literature [18,43,44]. The known sex differences in IGD prevalence may be attributable to the sex differences in maladaptive cognitions (eg, males demonstrated stronger perceived overvaluation of gaming rewards) [43], gaming motivations (eg, males tended to game for entertainment while females were more likely to game for social purposes) [45], and neurobiological functions (eg, males exhibited weaker control over game-elicited cravings) [46-48]. In addition, self-reported poor household financial situation is an indicator of low socio-economic status, which was associated with a variety of addictive behaviors, including IGD [18,44]. Such highlights that IGD intervention programs among university students may target these high-risk subgroups.

This study was the first to explore the differential associations between guilt and shame proneness and IGD. Interestingly, shame proneness in both cognitive and behavioral domains was positively associated with IGD, while guilt proneness in both domains was negatively associated with IGD. These results corroborate previous findings regarding the outcomes of internet addiction and problematic social media use [21-23]. Similar findings were also reported regarding other addiction problems. For instance, shame proneness was positively associated with various addiction outcomes of substance use problems, including tobacco, alcohol, and drugs, compulsive shopping, overeating, and compulsive sexual activities [20,49,50], while guilt proneness was negatively associated with alcohol use problems [49]. The emotion of shame is often internalized and related to one’s core sense of self [12,16]. Individuals prone to shame tend to have low self-esteem and self-worth [51] and adopt avoidant coping strategies [52], as indicated by the respective cognitive (ie, negative self-evaluation) and behavioral (ie, withdrawal action tendencies) domain of shame proneness in this study, both of which were positively associated with IGD [18,53,54]. In contrast, the emotion of guilt tends to focus on specific behaviours [12]. Individuals prone to guilt are often action-oriented and try to fix their mistakes, leading to adaptive coping strategies (eg, problem-solving behaviors) [12,20], and such might lower their likelihood of developing IGD. Notably, a previous study reported inconsistent results that interpersonal guilt was positively associated with IGD [24]. Conceptually, interpersonal guilt reflects specific actions that affect others in social interactions [24] while guilt proneness indicates a consistent, reparative tendency to experience guilt across various situations [19]. It is possible that, unlike guilt proneness, guilt derived from social interactions specifically might increase avoidant behaviors like internet gaming, which was in general considered an alternative to compensate for offline social interactions [55]. It is also likely that interpersonal guilt may become overly strong and generate maladaptive impacts, as adolescents tend to put greater value on interpersonal relationships while often addressing them in less favorable ways (eg, indulging in maladaptive thoughts and increasing risky behaviors such as internet gaming) [56]. Such speculations should be verified in future studies.

This study further confirmed the positive associations between emotional dysregulation of rumination, catastrophizing, and self-blame and IGD. Previous theoretical and empirical studies generally found that emotional dysregulation strategies would lead to unfavorable behavioral outcomes including IGD [26,30,31]. In addition, this study was novel in revealing the mediation effects of these emotional dysregulation strategies between guilt and shame proneness in both domains and IGD, with moderate mediation effect sizes (over 20% for guilt proneness and close to 50% for shame proneness). The partial mediation effects indicate that there might be other variables explaining the associations between shame/guilt proneness and IGD. For instance, shame proneness may affect self-isolation [57], which would in turn reinforce problematic gaming behaviors, increasing the reliance on virtual communities to gain social interactions and social acceptance [55].

### Recommendations

Given the present findings, shame proneness in both cognitive and behavioral domains should be mitigated in IGD interventions, which may also be effective in reducing emotional dysregulation. To address cognitive shame, fostering self-compassion and positive self-evaluation may be effective via self-compassion training (eg, teaching individuals how to treat themselves with kindness and avoid self-criticism) [58], cognitive-behavioral therapy (eg, helping individuals to challenge and reframe negative self-evaluation) [59], identifying shame triggers, and building self-worth. In contrast, provisions of training on adaptive coping strategies may be useful in modifying behavioral shame. In addition to behavioral activation of meaningful activities (eg, healthy hobbies and social interactions) and gradual exposures to social contexts where individuals could receive positive feedback to rebuild trust and reduce withdrawal, mindfulness-based stress reduction was shown effective in helping people to stay present and engage with their feelings without retreating into avoidant behaviors [59]. Furthermore, it may be effective to shift from shame toward adaptive guilt via positive reframing, which guides individuals to reframe self-judgments and personal failure as behavioral missteps rather than inherent flaws of personal identity [25]. Moreover, modifications to emotional dysregulation may be effective in reducing the risk of IGD directly and indirectly weakening the positive association between shame proneness (or enlarging the negative association involving guilt proneness) and IGD. Potentially effective approaches included cognitive-behavioral therapy [60], mindfulness techniques [61], acceptance and commitment therapy [62], and self-compassion training [63], which aims to foster cognitive flexibility and adaptive emotional regulation, helping individuals to break from maladaptive thought patterns.

### Limitations

This study was subject to several limitations. First, due to the cross-sectional nature of this study, causal and temporal inferences cannot be established. Future longitudinal and intervention studies are needed to verify the findings. Second, although this multicenter survey was conducted in seven cities covering the main geographical areas of China, the generalization of the results to other regions should be made cautiously and take sociocultural differences into consideration. For instance, Chinese culture emphasizes collective harmony and public image [64], which may strengthen the association between shame proneness and emotional dysregulation or IGD as shown in our sample. In contrast, in Western contexts where individualism and open emotional expression are more accepted [65], guilt proneness may function more adaptively or be less stigmatized, leading to weaker or even inverse associations with emotional dysregulation/IGD. Future studies are warranted to investigate these speculations from an ecological perspective. Third, this study comprised solely Chinese undergraduate students, which limits the generalizability of the findings to other populations, such as adolescents, older adults, or individuals outside academic settings. As the self-conscious emotions of guilt and shame develop early in life and may manifest differently across developmental stages [15], future studies should verify the current findings in younger or other age groups. Fourth, as this survey was self-administered, reporting bias may exist, including social desirability bias related to socially desirable responses (eg, underreporting IGD symptoms that were considered less socially desirable) and recall bias (eg, responding to questions with a long time frame such as past 12 mo). Fifth, this study used the 9-item DSM-5 IGD Checklist to assess IGD. Although its reliability and validity have been established in Chinese adults [34,35], the prevalence of IGD may be inflated in comparison to the ICD-11 criteria of gaming disorder [66]. Last, as aforementioned, there might be other mediation mechanisms between guilt and shame proneness and IGD that were not investigated in this study and should be investigated in future studies.

### Conclusions

This multicenter study observed a relatively high prevalence of IGD among medical undergraduate students in seven cities of China. Based on the SEM findings, this study revealed the differential associations between guilt and shame proneness and IGD. Shame proneness in both cognitive and behavioral domains might increase the risk of IGD, while guilt proneness in both domains might reduce the risk of IGD. Furthermore, emotional dysregulation of rumination, catastrophizing, and self-blame partially mediated the associations between guilt/shame proneness and IGD. These findings enhance the understanding of the associations between personality traits and IGD, as well as related mechanisms.

## Supplementary material

10.2196/74052 Multimedia Appendix 1 Questionnaire in English.

10.2196/74052 Multimedia Appendix 2 Questionnaire in Chinese.

10.2196/74052 Multimedia Appendix 3 Correlations among the key variables.

## Abbreviations:

CFI: Comparative Fit Index
DSM-5: The 5^th^ Edition of the Diagnostic and Statistical Manual of Mental Disorders
ICD-11: The 11^th^ Revision of the International Classification of Diseases
IGD: Internet gaming disorder
NBE: Negative behavior-evaluations
NSE: Negative self-evaluations
ORc: Crude odds ratio
RMSEA: Root Mean Square Error of Approximation
SEM: Structural equation modeling
SRMR: Root Mean Squared Residual
