# Supplementary material for: Emotional Dysregulation-Mediated Associations Between Guilt Proneness, Shame Proneness, and Internet Gaming Disorder Among Chinese University Students: Cross-Sectional Survey
Source: J Med Internet Res. 2025 Sep 5;27:e74052. doi: 10.2196/74052 (PMC12426564; doi:10.2196/74052)
Supplement: Multimedia Appendix 1 [file jmir-v27-e74052-s001.docx]

**English Questionnaire**

**Internet gaming disorder**

| Please indicate whether you have had the following symptoms in the past 12 months | No | Yes |
| --- | --- | --- |
| 1. Preoccupation with internet games (you think about previous gaming activity or anticipate playing the next game; internet gaming becomes the dominant activity in daily life) | 0 | 1 |
| 1. When you stop gaming or reduce gaming time, you feel irritable, anxious, or even sad | 0 | 1 |
| 1. You need to spend increasing amounts of time engaged in internet games to gain satisfaction or fun | 0 | 1 |
| 1. Unsuccessful attempts to control the participation in internet games | 0 | 1 |
| 1. Loss of interests in previous hobbies and entertainment as a result of, and with the except of, internet games | 0 | 1 |
| 1. Continued excessive use of internet games despite knowledge of psychosocial problems | 0 | 1 |
| 1. Has deceived family members or others regarding the amount of internet gaming | 0 | 1 |
| 1. Use of internet games to escape or relieve a negative mood (e.g., feelings of helplessness, guilt, anxiety) | 0 | 1 |
| 1. Has jeopardized or lost a significant relationship, job, or educational or career opportunity because of participation in internet games | 0 | 1 |

**Guilt/shame proneness**

In this questionnaire, you will read about situations that people are likely to encounter in

day-to-day life, followed by common reactions to those situations. As you read each scenario, try to imagine yourself in that situation. Then indicate the likelihood that you would react in the way described.

| 1  Very unlikely | 2  Unlikely | 3  Slightly unlikely | 4  About 50% unlikely | 5  Slightly likely | 6  Likely | 7  Very likely |
| --- | --- | --- | --- | --- | --- | --- |
| 1. After realizing you have received too much change at a store, you decide to keep it because the salesclerk doesn’t notice. What is the likelihood that you would feel uncomfortable about keeping the money? | | | | | | |
| 1. You are privately informed that you are the only one in your group that did not make the scholarship because you skipped too many days of school. What is the likelihood that this would lead you to become more responsible about attending school?？ | | | | | | |
| 1. You rip an article out of a journal in the library and take it with you. Your teacher discovers what you did and tells the librarian and your entire class. What is the likelihood that this would make you would feel like a bad person? | | | | | | |
| 1. After making a big mistake on an important project at work in which people were depending on you, your teacher criticizes you in front of all your classmates. What is the likelihood that you would feign sickness and leave work? | | | | | | |
| 1. You reveal a friend’s secret, though your friend never finds out. What is the likelihood that your failure to keep the secret would lead you to exert extra effort to keep secrets in the future? | | | | | | |
| 1. You give a bad presentation at work. Afterwards your teacher tells your classmates it was your fault that your team project did not win the award. What is the likelihood that you would feel incompetent? | | | | | | |
| 1. A friend tells you that you boast a great deal. What is the likelihood that you would stop spending time with that friend? | | | | | | |
| 1. Your home is very messy and unexpected guests knock on your door and invite themselves in. What is the likelihood that you would avoid the guests until they leave? | | | | | | |
| 1. You secretly commit a felony. What is the likelihood that you would feel remorse about breaking the law? | | | | | | |
| 1. You successfully exaggerate your losses to gain sympathy or compensation. Months later, your lies are discovered and you are blamed. What is the likelihood that you would think you are a despicable human being? | | | | | | |
| 1. You strongly defend a point of view in a discussion, and though nobody was aware of it, you realize that you were wrong. What is the likelihood that this would make you think more carefully before you speak? | | | | | | |
| 1. You take school supplies home for personal use and are caught by your teacher. What is the likelihood that this would lead you to quit school? | | | | | | |
| 1. You make a mistake in a team project and find out a classmate is blamed for the error. Later, your classmate confronts you about your mistake. What is the likelihood that you would feel like a coward? | | | | | | |
| 1. At a classmate’s housewarming party, you spill red wine on their new cream-colored carpet. You cover the stain with a chair so that nobody notices your mess. What is the likelihood that you would feel that the way you acted was pathetic? | | | | | | |
| 1. While discussing a heated subject with friends, you suddenly realize you are shouting though nobody seems to notice. What is the likelihood that you would try to act more considerately toward your friends? | | | | | | |
| 1. You lie to people but they never find out about it. What is the likelihood that you would feel terrible about the lies you told? | | | | | | |

**Emotional dysregulation**

|  | Almost never | Rarely | Occasionally | Frequently | Almost always |
| --- | --- | --- | --- | --- | --- |
| 1. I often think about how I feel about what I have experienced | 1 | 2 | 3 | 4 | 5 |
| 1. I am preoccupied with what I think and feel about what I have experienced | 1 | 2 | 3 | 4 | 5 |
| 1. I keep thinking about how terrible it is what I have experienced | 1 | 2 | 3 | 4 | 5 |
| 1. I continually think how horrible the situation has been | 1 | 2 | 3 | 4 | 5 |
| 1. I feel that I am the one who is responsible for what has happened | 1 | 2 | 3 | 4 | 5 |
| 1. I think that basically the cause must lie within myself | 1 | 2 | 3 | 4 | 5 |

**Background information**

a. Gender: □_1_ Male □_2_ Female

b. Age: ______ years

c. Year of study: □_1_ Year 1 □_2_ Year 2 □_3_ Year 3 □_4_ Year 4 □_5_ Year 5

c2. Study major: □_1_ Clinical medicine □_2_ Basic Medicine □_3_ Public Health

□_4_ Oral medicine (stomatology) □_5_ Medical imageology □_6_ Nursing □_7_ Pharmacy

□_8_ Traditional Chinese Medicine □_9_ Forensic medicine □_10_ Biomedicine

□_11_ Others________

d. Location of hometown: □_1_ Local city (where the university is located)

□_2_ Other cities in the university province □_3_ Other provinces

e. Household financial situation:

□_1_ Very good □_2_ Good □_3_  Moderate □_4_ Poor □_5_ Very poor
