# Supplementary material for: Emotional Dysregulation-Mediated Associations Between Guilt Proneness, Shame Proneness, and Internet Gaming Disorder Among Chinese University Students: Cross-Sectional Survey
Source: J Med Internet Res. 2025 Sep 5;27:e74052. doi: 10.2196/74052 (PMC12426564; doi:10.2196/74052)
Supplement: Multimedia Appendix 2 [file jmir-v27-e74052-s002.docx]

**中文版问卷（Chinese questionnaire）**

**网络游戏障碍**

| 请判断你在过去**12**个月是否存在以下的情况，然后根据真实情况进行回答。 | 无 | 有 |
| --- | --- | --- |
| **a** 你脑海里面充满关于网络游戏的事情（例如经常回想上次的游戏或者期望玩下一个游戏，游戏成为你日常生活中的主要活动） | 0 | 1 |
| **b** 当你要停止或者减少玩网络游戏时，会觉得烦躁、焦虑，或者甚至感到难过 | 0 | 1 |
| **c** 你感到自己需要用越来越多的时间去玩网络游戏，才可以获得满足感或者快乐 | 0 | 1 |
| **d** 你曾经多次尝试限制自己玩网络游戏的程度但未成功 | 0 | 1 |
| **e** 由于热衷于网络游戏，让你对以前其他的爱好及消遣活动失去兴趣 | 0 | 1 |
| **f** 即使你知道网络游戏玩得太多会引起心理或者社交问题，你仍继续玩 | 0 | 1 |
| **g** 你对其他人欺瞒你对网络游戏的热衷程度 | 0 | 1 |
| **h** 你试过将玩网络游戏作为逃避或者舒缓负面情绪（例如无助感、内疚感、焦虑感）的途径 | 0 | 1 |
| **i** 你因为热衷于网络游戏而危害到或者失去一段重要的关系或升学的机会 | 0 | 1 |

**内疚/羞耻倾向**

下面是一些情境故事，故事描述的是一些你在学习和生活中可能会遇到的事情，请仔细阅读，并想象你自己正在经历故事中所描述的事情，然后判断每种原因解释是否真实的可能性大小。

| 1  非常不可能 | 2  不可能 | 3  有点不可能 | 4  一半一半 | 5  有点可能 | 6  可能 | 7  非常可能 |
| --- | --- | --- | --- | --- | --- | --- |
| 1. 你在超市买完东西付款后，发现收银员找给你的零钱比实际应该找的数目多很多。由于收银员没有发现，你决定拿走这些零钱。那么，你因为拿走这些零钱而感到不舒服的可能性有多大？ | | | | | | |
| 1. 你被私下告知你是相熟同学中唯一一个因为逃课太多而没有评上奖学金的人，你因为这个事情而对自己的学业变得更有责任心的可能性有多大？ | | | | | | |
| 1. 你从图书馆的书里撕下几页内容私自保存，老师发现后告诉了图书馆管理员和全班同学。你因为这件事情而觉得自己是个坏人的可能性有多大？ | | | | | | |
| 1. 你在学校带领几个同学完成一个重要的集体项目时犯了一个大错误，负责老师因此在所有同学面前批评了你。你因为这样而装病退出项目的可能性有多大？ | | | | | | |
| 1. 你将朋友的秘密告诉了别人。虽然你的朋友永远也不会发现这个事情，但你因为自己这次没有守住秘密而在未来更加努力去守住其他秘密的可能性有多大？ | | | | | | |
| 1. 你在集体项目做报告时表现很差。事后老师告诉同学是因为你的表现而使项目没有评上奖。你因此觉得自己没有能力的可能性有多大？ | | | | | | |
| 1. 一个朋友跟你说你吹牛吹得很离谱，你因此不和这个朋友来往的可能性有多大？ | | | | | | |
| 1. 你的家里很乱，这时候有未经邀约的客人上门拜访，你假装自己不在家里从而回避他/她进门的可能性有多大？ | | | | | | |
| 1. 你犯下了不为人知的重罪（如盗窃），你因犯法而感到悔恨的可能性有多大？ | | | | | | |
| 1. 你在某件事情上成功地夸大了自己的损失而得到了别人的同情或赔偿。几个月后，别人发现了你撒谎并对你进行指责。你因此觉得自己是个卑鄙的人的可能性有多大？ | | | | | | |
| 1. 你在某次和别人的讨论中强烈地捍卫自己的观点，事后你意识到自己的观点是错误的。虽然其他人没有发现这个错误，但你因此在下次发言前更加仔细地思考自己观点的可能性有多大？ | | | | | | |
| 1. 你将学校的公用物品带回宿舍作为自己的私人物品使用。老师发现了这个事情，你因此而退学的可能性有多大？ | | | | | | |
| 1. 你在集体项目中犯了错误，但发现你的同学因为这个错误而受到指责。事后，这个同学因为这件事情和你对质，你觉得自己没有承担责任、是个懦夫的可能性有多大？ | | | | | | |
| 1. 去同学家里聚会的时候，你不小心将有色饮料洒在同学家里的白色沙发上，你拿一个抱枕盖住这个污渍以防别人发现。你觉得自己的这个行为很差劲的可能性有多大？ | | | | | | |
| 1. 当你与朋友讨论一个热门话题的时候，你突然意识到自己很激动、嗓门很大。虽然别人都没有注意到，但你因此尝试更体贴地对待你的朋友的可能性有多大？ | | | | | | |
| 1. 你对别人撒了谎。虽然他们永远都不会发现那些是谎言，但你因为自己撒谎感到糟糕的可能性有多大？ | | | | | | |

**情绪调节**

|  | 几乎从不 | 偶尔 | 有时 | 经常 | 几乎总是 |
| --- | --- | --- | --- | --- | --- |
| 1. 我常常会想对我已经经历的事是怎样的感觉 | 1 | 2 | 3 | 4 | 5 |
| 1. 我沉迷于我已经经历的事的感觉和想法 | 1 | 2 | 3 | 4 | 5 |
| 1. 我不断地想我经历的事情是多么的可怕 | 1 | 2 | 3 | 4 | 5 |
| 1. 我不断的想这个事情是多么的可怕 | 1 | 2 | 3 | 4 | 5 |
| 1. 我感到我应该被责备 | 1 | 2 | 3 | 4 | 5 |
| 1. 我想事情的基本原因在我自己 | 1 | 2 | 3 | 4 | 5 |

**背景信息**

a. 请问你的性别是？ □_1_男 □_2_女

b. 年龄：______ 周岁

c. 请问你的年级是? □_1_ 大一 □_2_ 大二 □_3_ 大三 □_4_ 大四 □_5_ 大五

c2. 请问你的专业是？ □_1_ 临床医学 □_2_ 基础医学 □_3_ 公共卫生与预防医学 □_4_ 口腔医学 □_5_ 医学影像学 □_6_ 护理学 □_7_药学 □_8_中医学 □_9_法医学 □_10_ 生物医学 □_11_ 其他________

d. 你的生源地是：□_1_本市（大学所在城市） □_2_ 本省的其他地区 □_3_ 其他省

e. 你家里的经济状况: □_1_非常好 □_2_较好 □_3_一般 □_4_较差 □_5_非常差
