# Supplementary material for: Emotional Dysregulation-Mediated Associations Between Guilt Proneness, Shame Proneness, and Internet Gaming Disorder Among Chinese University Students: Cross-Sectional Survey
Source: J Med Internet Res. 2025 Sep 5;27:e74052. doi: 10.2196/74052 (PMC12426564; doi:10.2196/74052)
Supplement: Multimedia Appendix 3 [file jmir-v27-e74052-s003.docx]

Table S1 Correlations among the key variables

|  | 1 | 2 | 3 | 4 | 5 | 6 | 7 |
| --- | --- | --- | --- | --- | --- | --- | --- |
| 1. Guilt-NBE | - |  |  |  |  |  |  |
| 2. Guilt-repair | 0.88  (0.87, 0.88)  (*P< .001*) | - |  |  |  |  |  |
| 3. Shame-NSE | 0.72  (0.71, 0.73)  (*P< .001*) | 0.79  (0.78, 0.80)  (*P< .001*) | - |  |  |  |  |
| 4. Shame-withdrawal | 0.41  (0.40, 0.42)  (*P< .001*) | 0.40  (0.39, 0.41)  (*P< .001*) | 0.55  (0.54, 0.56)  (*P< .001*) | - |  |  |  |
| 5. Rumination | -0.26  (-0.28, -0.24)  (*P< .001*) | -0.27  (-0.29, -0.25)  (*P< .001*) | 0.30  (0.29, 0.32)  (*P< .001*) | 0.23  (0.21, 0.26)  (*P< .001*) | - |  |  |
| 6. Catastrophizing | -0.05  (-0.07, -0.05)  (*P< .001*) | -0.04  (-0.06, -0.04)  (*P< .001*) | 0.17  (0.15, 0.19)  (*P< .001*) | 0.27  (0.25, 0.29)  (*P< .001*) | 0.62  (0.62, 0.64)  (*P< .001*) | - |  |
| 7. Self-blame | -0.12  (-0.14, -0.12)  (*P< .001*) | -0.10  (-0.12, -0.10)  (*P< .001*) | 0.23  (0.21, 0.26)  (*P< .001*) | 0.22  (0.20, 0.24)  (*P< .001*) | 0.56  (0.56, 0.58)  (*P< .001*) | 0.71  (0.70, 0.71)  (*P< .001*) | - |
| 8. IGD | -0.03  (-0.04, -0.02)  (*P= .02*) | -0.04  (-0.03, -0.01)  (*P= .001*) | 0.03  (0.01, 0.05)  (*P= .01*) | 0.09  (0.07, 0.11)  (*P< .001*) | 0.13  (0.11, 0.15)  (*P< .001*) | 0.19  (0.17, 0.21)  (*P< .001*) | 0.21  (0.19, 0.23)  (*P< .001*) |

Note. NBE = Negative behavior-evaluations; NSE = Negative self-evaluations; IGD = Internet gaming disorder. Correlation coefficients and their 95% confidence intervals were reported. Spearman correlation analyses were conducted between IGD and other key variables, while Pearson correlation analyses were conducted among variables of guilt/shame proneness and emotional dysregulation.
